# Validation of a 3-h Sampling Interval to Assess Variability in Cytochrome P450 3A Phenotype and the Impact of Induction and Mechanism-Based Inhibition Using Midazolam as a Probe Substrate

**DOI:** 10.3389/fphar.2019.01120

**Published:** 2019-09-27

**Authors:** Madelé van Dyk, Asha J. Kapetas, Ashley M. Hopkins, A. David Rodrigues, Manoli Vourvahis, Michael J. Sorich, Andrew Rowland

**Affiliations:** ^1^College of Medicine and Public Health, Flinders University, Adelaide, SA, Australia; ^2^ADME Sciences, Medicine Design, Pfizer Inc, Groton, CT, United States; ^3^Clinical Pharmacology, Global Product Development, Pfizer Inc, New York, NY, United States

**Keywords:** clarithromycin, cytochrome P450 3A4, induction, mechanism-based inhibition, midazolam, phenotyping studies, rifampin

## Abstract

**Background:** Drug probe phenotyping is used extensively in academic and industry research to evaluate cytochrome P450 (CYP) phenotype in order to account for sources of between- and within- subject variability in metabolic clearance. In terms of application, CYP3A is the most important drug metabolizing enzyme the most frequently studied. Currently, phenotyping studies for CYP3A involve the administration of midazolam and collection of timed blood samples up to 24-48 hours in order to determine an area under the plasma concentration time curve (AUC). The key challenge that limits the use of midazolam-based phenotyping for CYP3A in academic research settings and preclude the use of this approach in a clinical setting is the logistical burden of collecting frequent blood samples for up to 48 h post dose following the administration of a probe drug ± an interacting drug.

**Aim:** The current study sought to validate if a reduced sampling interval could be used to accurately define both between-subject variability in CYP3A phenotype and the magnitude of changes in CYP3A activity due to either induction or mechanism-based inhibition.

**Methods:** The area under the curve (AUC) for midazolam was assessed under baseline, induction (7 days rifampin, 300 mg daily) and, following a washout period of 4 days, mechanism based inhibition (3 days clarithromycin, 250 mg daily) conditions in a cohort of 30 health males. The capacity of normalized reduced sampling interval AUCs measured over 0 to 1, 0 to 2, 0 to 3, and 0 to 4 h to accurately define the AUC_0-6_ was evaluated with respect to precision (R2 for correlation), bias (slope of normalized correlation), agreement (Bland Altman analysis) and proportional bias (linear regression of Bland Altman parameters).

**Results:** Robust concordance was observed between the AUC calculated from PK collection intervals of 0 to 3 and 0 to 6 h in terms of both the measurement of between-subject variability in midazolam AUC and changes in midazolam AUC due to induction and mechanism-based inhibition of CYP3A4.

**Conclusion:** On this basis, it is proposed that a 3-h assessment of midazolam AUC (AUC_0-3_) represents a viable strategy to reduce the logistical burden associated with the assessment of CYP3A phenotype.

## Introduction

*In vivo* drug probe phenotyping is the current gold standard approach to evaluate cytochrome P450 (CYP) phenotype in order to account for sources of between- and within- subject variability in metabolic clearance (Rowland et al., 2016). This approach, which is used extensively in academic and industry research, involves the administration of a drug that is a selective substrate for an enzyme of interest and subsequent collection of timed blood samples, typically over 24–48 h, in order to determine the area under the plasma drug concentration time curve (AUC) (Ryu et al., 2007). By assessing the probe drug’s AUC across different populations (e.g., racial/ethic groups, genotype) or before and after the administration of an interacting drug (e.g., potential enzyme inducer), it is possible to define the impact of an interacting drug or a population characteristic on the phenotype of the target CYP.

In terms of the application, CYP3A4 and 3A5 (collectively referred to as CYP3A) are the most clinically important drug metabolizing enzymes thus the most frequently investigated in phenotyping studies (Zanger and Schwab, 2013). By way of example, in drug development when a new chemical entity (NCE) is identified in pre-clinical testing as a potential ‘perpetrator’ of a metabolic drug-drug interaction (DDI) involving CYP3A, a phenotyping study is required to assess the change in AUC for a sensitive CYP3A substrate pre-/post-dosing of the NCE (Rowland et al., 2018; Kapetas et al., 2019). When evaluating CYP3A phenotype, midazolam is the most commonly used probe and is recommended as such in US Food and Drug Administration (FDA) guidance (US Food and Drug Administration, 2017). Midazolam is an intermediate hepatic clearance (CL_H_ = 27 L/h) drug that is extensively metabolized by CYP3A4, CYP3A5, and CYP3A7 (Williams et al., 2002) making it an ideal probe to determine both hepatic and intestinal CYP3A phenotypes when administered orally (Thummel et al., 1994a; Thummel et al., 1994b; Thummel et al., 1996).

The key challenge that limits the use of phenotyping for CYP3A in academic research settings and preclude the use of this approach in a clinical setting is the logistical burden of collecting blood samples for up to 24–48 h post dose following the administration of a probe drug with or without an interacting drug (Rowland et al., 2017). In order to circumvent this limitation, several endogenous biomarker based strategies have been proposed to characterize CYP3A phenotype (Bjorkhem-Bergman et al., 2013; Mårde Arrhén et al., 2013; Rodrigues and Rowland, 2018; Rowland et al., 2019). While these strategies may ultimately replace the need to administer a drug probe, none have been adopted to date due to either inherent biological limitations (e.g., the complexity of steroid metabolism which involves multiple sequential, parallel or alternate metabolic pathways for the substrate (probe) synthesis, substrate metabolism and product metabolism) (Winters and Clark, 2003) or a current limited body of evidence (e.g., extracellular vesicle derived protein, *ex vivo* activity or mRNA biomarkers) (Rodrigues and Rowland, 2018). Similarly, several strategies including the administration of midazolam micro-dosing (Eap et al., 2004; Hohmann et al., 2015), evaluation of a plasma hydroxy-midazolam to midazolam metabolic ratios (Shih and Huang, 2002; Chaobal and Kharasch, 2005), and limited PK sampling strategies (Lee et al., 2006; Katzenmaier et al., 2010; Mueller and Drewelow, 2013; Tai et al., 2013; Masters et al., 2015; Yang et al., 2018) have been proposed, but have not been widely adopted. Of these strategies, to date only the use of a 75 µg oral micro-dose has been rigorously verified (Hohmann et al., 2015). The notable limitation with this micro-dose strategy that continues to limit its application is that it requires access to a sufficiently sensitive analytical platform capable of quantifying femtomolar midazolam concentrations in plasma (Burhenne et al., 2012; Hohmann et al., 2015). Likewise, the use of a single hydroxy-midazolam to midazolam concentration ratio collected at a specific time point as a surrogate for midazolam AUC has largely been discredited as a viable option given the poor concordance of this metabolic ratio with midazolam exposure (Lee et al., 2006).

Similarly, the performance of existing limited sampling protocols (Kim et al., 2002; Lee et al., 2006; Katzenmaier et al., 2010; Mueller and Drewelow, 2013; Tai et al., 2013; Masters et al., 2015; Yang et al., 2018) have only been verified with respect to the concordance of describing between-subject variability in CYP3A phenotype, in cases with sporadic sampling under baseline, inhibited or induced conditions. To date, no reduced sampling protocol has been verified with respect to performance in terms of defining changes in CYP3A phenotype due to either inhibition or induction of catalytic activity within a single cohort of individuals. Notably in this regard, the prior study of Katzenmaier et al. (2010) proposed a sampling interval (2–4 h) that fails to capture the midazolam absorption and distribution phases (hence cannot account for differences in C_max_). The importance of this omission being that the time course and magnitude of CYP3A induction and mechanism-based inhibition in the liver and intestine are not equivalent (Kapetas et al., 2019), meaning that changes in CYP3A phenotype described by midazolam AUC cannot be robustly defined unless the sampling protocol captures both C_max_ and a sufficient window of the elimination phase. Similarly, in many cases the focus has been on minimizing the number of sample draws rather than the duration over which the samples are collected (Mueller and Drewelow, 2013; Tai et al., 2013), by way of example Mueller et al. (Mueller and Drewelow, 2013) demonstrated that midazolam AUC can be accurately defined using only 4 data-points, albeit over an 8-h interval. The current study sought to validate if a reduced sampling interval that retains rich sampling during the first hour (i.e. focus on shorter timeframe, not fewer samples), could be used to accurately define both between-subject variability in CYP3A phenotype as well as the magnitude of change in CYP3A activity due to either induction or mechanism-based inhibition.

## Methods

### Study Protocol

**Figure 1:**
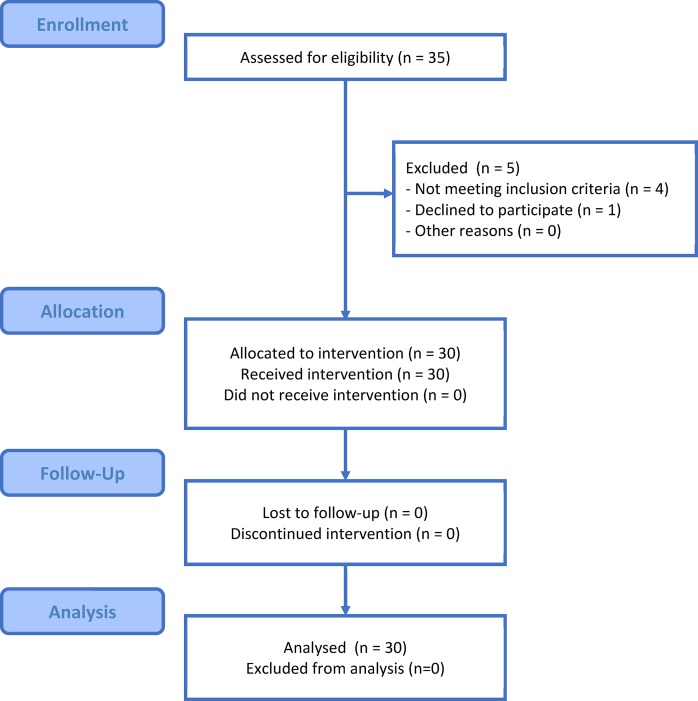
CONSORT diagram showing progression of healthy participants through the trial.

EPOK-15 is a single-center, open-label, single-sequence metabolic phenotyping study (van Dyk et al., 2018; Rowland et al., 2019). The study protocol was approved by the Southern Adelaide Clinical Human Research Ethics Committee (SAHREC 11.15), and written informed consent was obtained from each participant prior to study enrolment (**Figure 1**). The study was conducted according to the principles stated in the Declaration of Helsinki, was compliant with CPMP/ICH/135/95 GCP standards and is registered with the Australian New Zealand Clinical Trials Registry (ACTRN 12614001289606).

Midazolam pharmacokinetics following the administration of a 1 mg oral midazolam dose was assessed in a cohort of healthy males (n = 30; **Table 1**) at baseline (Day 1), following a 7 day course of rifampin (Induction phase; Day 8) and following a 3 day course of immediate release clarithromycin (Mechanism-based inhibition phase; Day 15) with a four day washout period between the induction and mechanism-based inhibition phases. Blood samples collected prior to dosing and at 0.25, 0.5, 0.75, 1, 2, 3, 4 and 6 h post-dosing were centrifuged and plasma were stored at -80°C until analysis for the determination of midazolam concentrations. Participants self-administered rifampin (300 mg daily PO) and clarithromycin (250 mg twice daily PO) from days 1–8 and days 12–14, respectively.

**Table 1:** Summary of study participant demographics.

	Training cohort (n = 10)		Verification cohort (n = 20)	
	Mean	Range	Mean	Range
*Physiology*				
Age (years)	26.7	21 – 32	25.8	21 – 34
Height (cm)	1.76	1.65 – 1.82	1.76	1.64 – 1.93
Weight (kg)	72.2	62.7 – 84.3	76.9	57.2 – 108
BMI (kg/m^2^)	23.4	20.1 – 26.8	24.7	18.7 – 30.1
*Race*				
Caucasian (n)	7		12	
Asian (n)	3		8	
*CYP3A genotype*				
CYP3A4*1/*1 (n)	10		18	
CYP3A4*1/*22 (n)	0		2	
CYP3A5*3/*3 (n)	9		14	
CYP3A5*1/*3 (n)	1		6	

Physiological data are mean and range, CYP3A genotypes and race are number of patients.

### Sample Preparation and Analyses

The sample preparation and analysis for the determination of midazolam and 1-OH midazolam concentrations has been reported previously (van Dyk et al., 2018). Briefly, protein from 100 μl of plasma sample was precipitated using 300 μl of methanol containing 0.1% formic acid and 7.5 ng/ml d_6_-midazolam (assay internal standard). Samples were vortexed for 3 min, then centrifuged at 16,000 g for 5 min. Analytes in a 2.5 μl aliquot of the resulting supernatant were separated from the sample matrix by ultra-performance liquid chromatography (UPLC) performed on a Waters ACQUITY™ BEH C18 column (100 mm × 2.1 mm, 1.7 μm; Waters Corp., Milford, USA) using a Waters ACQUITY™ UPLC system. Column elutant was monitored by mass spectrometry, performed on a Waters Q-ToF Premier™ quadrupole, orthogonal acceleration time-of-flight tandem mass spectrometer operating in positive electron spray ionization (ESI+) mode. Selected ion data was extracted at the analyte [M+H]+ precursor m/z. Resulting pseudo-MRM spectra were analyzed using Waters TargetLynx™ software. Plasma analyte concentrations were determined by comparison of normalized peak areas in participant samples to those of external calibrators. As described previously, rifampin and clarithromycin plasma concentrations were also determined pre-midazolam dosing on day 8 and day 15, respectively (van Dyk et al., 2018).

### Data Analysis

Non-compartmental methods were used to calculate the area under the midazolam plasma concentration time curve (AUC_0-t_) for PK sampling intervals of 0–1, 0–2, 0–3, 0–4, and 0–6 h post midazolam dosing on study day 1 (baseline), day 8 (following a 7-day course of rifampin), and day 15 (following a 3-day course of clarithromycin). Similarly, the pharmacokinetic parameters maximal concentration (C_max_), time to maximal concentration (T_max_), elimination half life (t_1/2_), elimination rate constant (k) and full area under the curve (AUC_0-inf_) were determined using non-compartmental methods (Microsoft Excel, PK Functions for Microsoft Excel, Department of Pharmacokinetics and Drug Metabolism, Irvine, CA, USA). AUC_0-inf_ was calculated as AUC_0-6_ plus AUC_6-inf_, where AUC_6-inf_ (the extrapolated AUC) was calculated by dividing the final measured concentration (C_last_) the elimination rate constant (k_e_) determined over the period t_max_ to 6 h. In all cases the % extrapolated (AUC_6_inf_/AUC_0-inf_) was less than 20%.

The performance of test sampling intervals (AUC_0-1_, AUC_0-2_, AUC_0-3_ and AUC_0-4_) and the 1-OH midazolam to midazolam ratio measured at 3, 4, and 6 h were evaluated with respect to agreement of the normalized reduced sampling interval AUCs (or metabolite ratios) with the midazolam AUC_0-6_. Participant AUC data grouped by study phase was randomised 1:2 into training and validation data sets. The training dataset (n = 10 participants) was used to derive linear scaling factors (Equation 1) to normalize each of the AUC_0-1_, AUC_0-2_, AUC_0-3_ and AUC_0-4_ to the AUC_0-6_.

$${\text{AUC}}_{0-6}=\text{factor}\times {\text{AUC}}_{0-t}$$

where “factor” is the scaling factor determined for each sampling interval (**Table 4**), and “t” is the final sampling time for each interval (i.e., 1, 2, 3, or 4 h).

Validation datasets (n = 60) for midazolam AUC_0-1_, AUC_0-2_, AUC_0-3_ and AUC_0-4_ were normalized to AUC_0-6_ using the scaling factor derived from the respective training sets. Bland-Altman analyses were performed to evaluate the correlation between AUC_0-6_ and the normalized reduced sampling interval AUCs calculated from reduced PK sampling intervals. Linear regression was performed to assess proportional bias based on the influence of mean AUC (normalized reduced sampling interval AUC and AUC_0-6_) on the difference in AUC (normalized reduced sampling interval AUC versus AUC_0-6_).

The performance of each reduced sampling interval AUC was also evaluated with respect to the concordance of the change (Δ) in midazolam exposures due to induction and mechanism-based inhibition of CYP3A. Using the same validation data set described for the assessment of the performance of the absolute AUC, midazolam AUC ratios were calculated for induction (AUC_day8_/AUC_day1_) and inhibition (AUC_day15_/AUC_day1_). Bland-Altman analyses were performed to evaluate the correlation between the respective AUC ratios for the reduced sampling interval AUCs and the corresponding AUC_0-6_ ratio. Linear regression was performed to assess proportional bias based on the influence of mean of the normalized reduced sampling interval AUCs and AUC_0-6_ parameters on the difference between the normalized reduced sampling interval AUCs and the AUC_0-6_ parameters. Bland-Altman plots were created by plotting the difference between the normalized reduced interval AUC and the AUC_0-6_ on the y-axis and the mean of the reduced interval AUC and the AUC_0-6_ on the y-axis.

Criteria for the acceptance of a reduced sampling interval AUC in the validation dataset were:

- An R^2^ for the correlation of the test parameter (AUC or AUC ratio) with the AUC_0-6_ parameter of >0.85 with and without applying a forced zero intercept.
- A slope for the correlation of the test parameter with the AUC_0-6_ parameter in the range 0.9 to 1.1 when applying a forced zero intercept.
- A lack of statistical significance for a one sample *t*-test of the difference in test and AUC_0-6_ parameters versus 0.
- A lack of statistical significance for the linear regression of the effect of the mean of normalized reduced sampling interval AUC and AUC_0-6_ on the difference between the normalized reduced sampling interval AUC and the AUC_0-6_. That is to exclude a proportional bias in the dataset, and to ensure that the magnitude and direction of difference between the normalized reduced sampling interval AUC and the AUC_0-6_ is independent of the magnitude of the mean of these AUCs.

## Results

### Midazolam Exposure

**Figure 2:**
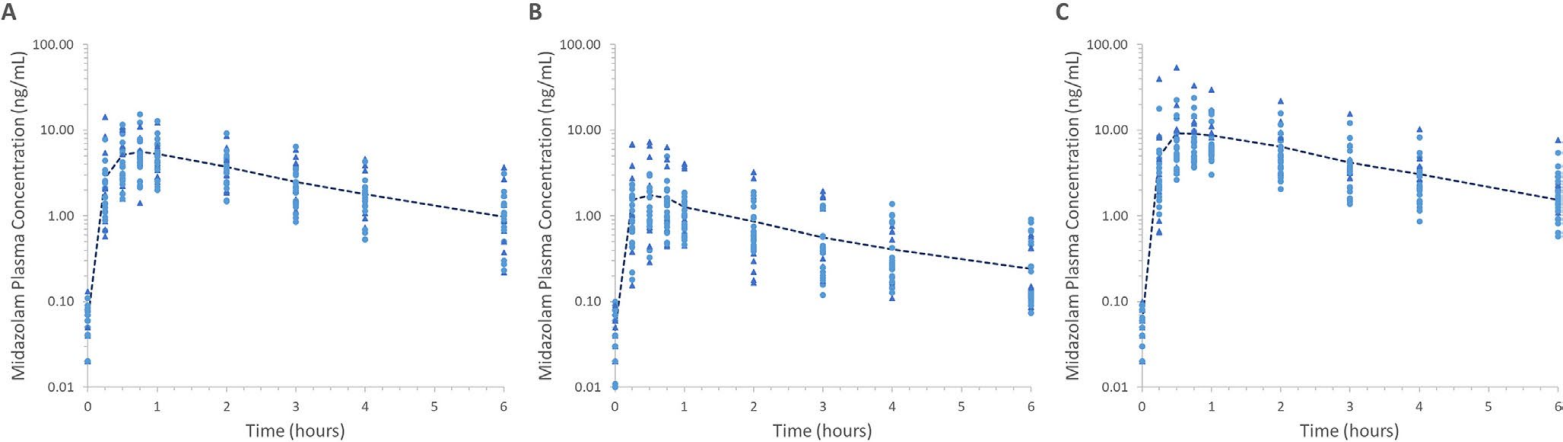
Midazolam plasma concentration time plots for baseline **(A)**, induction **(B)** and Mechanism-based inhibition **(C)** study phases. Triangles represent training dataset, circles represent verification dataset, dashed line represents mean profile for the full dataset.

A summary of the geometric mean (95% CI) midazolam AUC baseline, post- induction and post- mechanism-based inhibition are reported in **Table 2**. Additional pharmacokinetic parameters defining midazolam exposure for each study phase are reported in **Table 3**. The distribution of midazolam concentration time profiles within the training and verification datasets for each of the three study phases (baseline, induction and mechanism based inhibition) is shown in **Figure 2**. There were no statistically significant differences in parameter estimates between the full, training and validation data sets. Dosing of rifampin (300 mg daily) for 7 days resulted in mean 69% and 68% reductions in midazolam AUC_0-6_ and C_max_, respectively. Dosing of clarithromycin (250 mg twice daily) for 3 days resulted in mean 49% and 40% increases in midazolam AUC_0-6_ and C_max_, respectively.

**Table 2:** AUC values defining midazolam exposure in the training, verification and full cohorts.

Parameter	Study phase	Training cohort (n = 10)			Verification cohort (n = 20)			Full cohort (n = 30)		
		Geometric mean (ratio*)	95% CI		Geometric mean (ratio*)	95% CI		Geometric mean (ratio*)	95% CI	
			Lower	Upper		Lower	Upper		Lower	Upper
AUC_0-1_ (ng/ml/h)	Control	4.4	2.4	6.3	4	3	4.9	4.1	3.3	4.9
	Induced	2 (0.45)	0.6	3.5	1.2 (0.30)	0.9	1.5	1.5 (0.37)	1.0	2.0
	Inhibited	6.4 (1.45)	3.7	9	5.8 (1.45)	4.1	7.6	5.9 (1.44)	4.6	7.2
AUC_0-2_ (ng/ml/h)	Control	9	5.6	12.4	8.4	6.6	10.3	8.6	7.1	10.2
	Induced	3.4 (0.38)	1	5.9	2.1 (0.25)	1.6	2.6	2.6 (0.30)	1.7	3.4
	Inhibited	14 (1.56)	9.1	19	12.3 (1.46)	9	15.7	12.6 (1.47)	10.1	15.2
AUC_0-3_ (ng/ml/h)	Control	12.3	7.7	16.8	11.4	9	13.9	11.7	9.6	13.8
	Induced	4.6 (0.37)	1.2	8.1	2.8 (0.25)	2.1	3.4	3.4 (0.29)	2.2	4.5
	Inhibited	19.3 (1.57)	13	25.6	16.8 (1.47)	12.2	21.5	17.3 (1.48)	13.8	20.8
AUC_0-4_ (ng/ml/h)	Control	14.7	9.1	20.3	13.5	10.6	16.4	13.9	11.4	16.4
	Induced	5.7 (0.39)	1.4	10	3.3 (0.24)	2.5	4.2	4.1 (0.31)	2.7	5.5
	Inhibited	22.7 (1.54)	15.8	29.5	20.0 (1.48)	14.4	25.7	20.5 (1.49)	16.3	24.6
AUC_0-6_ (ng/ml/h)	Control	18.3	10.8	25.9	16.2	12.7	19.7	16.9	13.8	20.1
	Induced	7.2 (0.39)	1.5	12.9	4.4 (0.27)	3.1	5.7	5.3 (0.31)	3.4	7.2
	Inhibited	28.2 (1.54)	20.1	36.3	24.6 (1.52)	17.7	31.5	25.2 (1.49)	20.2	30.3
AUC_0-inf_ (ng/ml/h)	Control	24.8	11.8	37.8	20.2	15.7	24.6	21.6	16.7	26.4
	Induced	13.4 (0.54)	1.8	28.6	7.6 (0.38)	4.1	11.1	9.6 (0.44)	4.5	14.6
	Inhibited	50.6 (2.04)	22.3	78.9	31.6 (1.56)	22.7	40.5	38.1 (1.76)	27.5	48.7

*Ratio of induced (post- rifampin)/control parameter for ‘Induced’ and inhibited (post- clarithromycin)/control parameter for ‘inhibited’ study phases.

**Table 3:** Pharmacokinetic parameters defining midazolam concentration time profile in the training, verification and full cohorts.

Parameter	Study phase	Training cohort (n = 10)			Verification cohort (n = 20)			Full cohort (n = 30)		
		Geometric mean	95% CI		Geometric mean	95% CI		Geometric mean	95% CI	
			Lower	Upper		Lower	Upper		Lower	Upper
C_max_ (ng/ml)	Control	7	4.5	9.5	6.3	4.7	7.8	6.5	5.3	7.7
	Induced	2.8	0.8	4.9	1.7	1.2	2.2	2.1	1.4	2.8
	Inhibited	9.5	6	13	8.9	6.4	11.4	9.1	7.2	11.0
T_max_ (h)	Control	0.83	0.47	1.18	0.83	0.62	1.03	0.83	0.66	1.00
	Induced	0.55	0.33	0.77	0.65	0.46	0.85	0.62	0.47	0.76
	Inhibited	0.75	0.58	0.92	0.98	0.75	1.20	0.90	0.74	1.06
T_1/2_ (h)	Control	2.4	1.6	3.1	2.2	1.8	2.5	2.2	1.9	2.5
	Induced	1.5	1.0	2.5	1.5	1.2	2.8	1.3	0.9	2.2
	Inhibited	2.7	1.6	3.7	2.7	2.2	3.2	2.5	2.1	2.9
CL/F (L/h)	Control	55.7	36.6	74.8	62.1	47.2	77.1	60.0	48.8	71.2
	Induced	276	136	417	239	176	302	251	193	309
	Inhibited	28.6	18.3	38.9	42.9	31.2	54.7	38.1	29.6	46.6

### Capacity of Reduced Sampling Interval to Define Absolute Midazolam AUC

**Figure 3:**
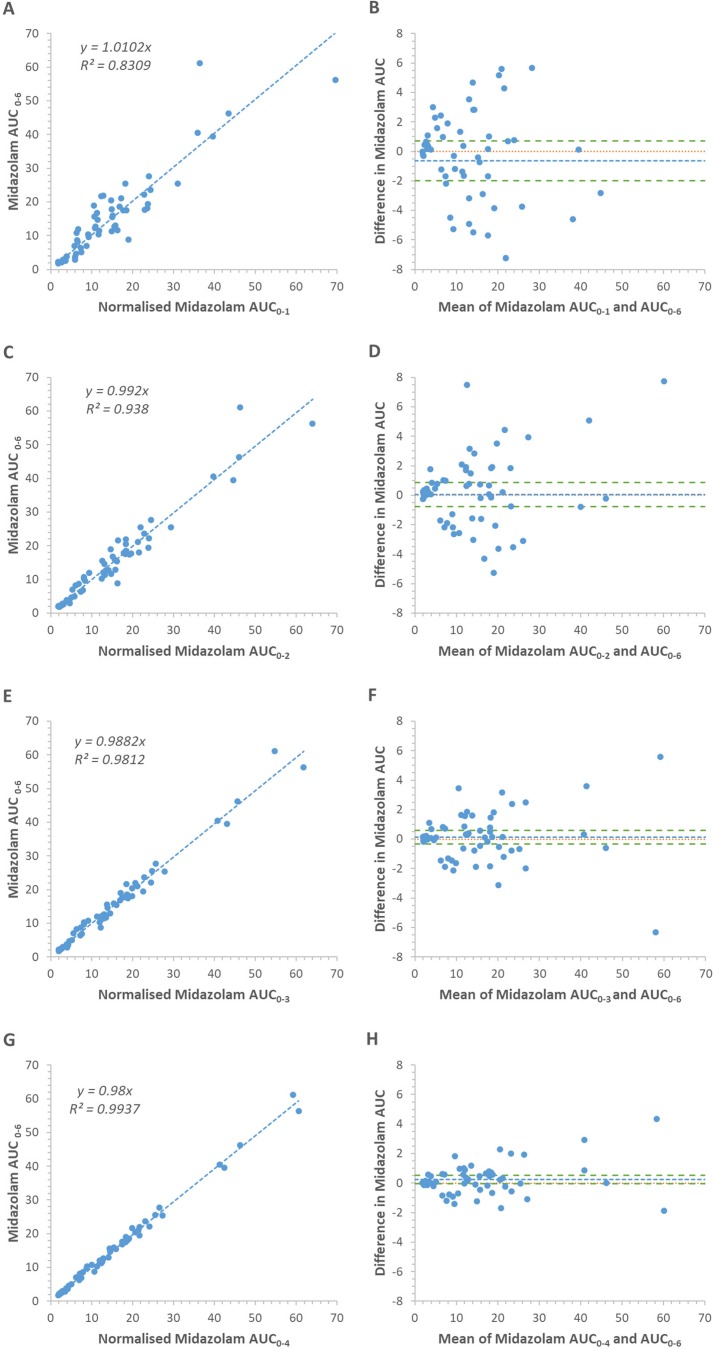
Concordance of midazolam exposure between normalized reduced sampling interval AUCs and AUC_0-6_ in the validation cohort. **(A**, **C**, **E**, and **G)** Correlation plots for the AUC_0-1_, AUC_0-2_, AUC_0-3_ and AUC_0-4_ sampling intervals respectively. **(B**, **D**, **F**, and **H)** Bland-Altman plots (mean of normalized reduced sampling interval AUCs and AUC_0-6_ parameters versus difference between normalized reduced sampling interval AUCs and AUC_0-6_ parameters) for the AUC_0-1_, AUC_0-2_, AUC_0-3_, and AUC_0-4_ sampling intervals, respectively. Lines describe the mean difference (blue line), 95% confidence interval around the mean difference (green lines) and the zero difference reference (orange line). Units: AUC; ng/mL/h.

**Figure 4:**
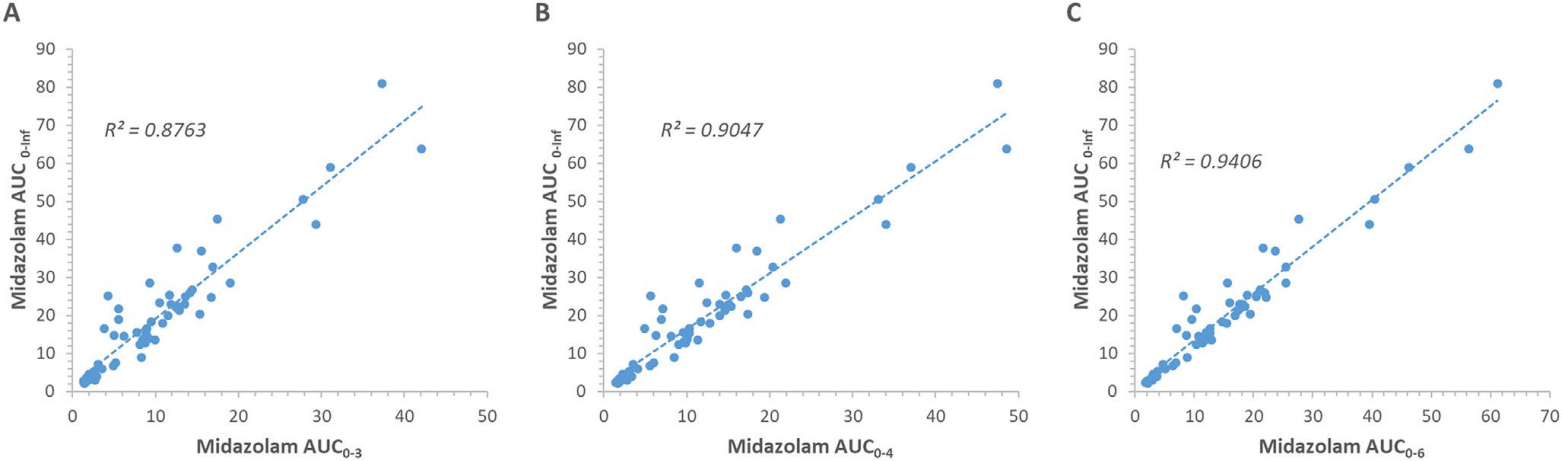
Concordance of midazolam exposure between AUC_0-3_, AUC_0-4_, and AUC_0-6_ and the full AUC (AUC_0-inf_) in the validation cohort. **(A**, **B**, **C)** Correlation plots for the AUC_0-3_, AUC_0-4_, and AUC_0-6_ sampling intervals, respectively. Units: AUC; ng/mL/h.

A training dataset (n = 10; 30 data points) was used to derive equations that normalized each reduced sampling interval AUC (AUC_0-1_, AUC_0-2_, AUC_0-3_, and AUC_0-4_) to midazolam AUC_0-6_ (**Table 4**). The concordance of each normalized reduced interval AUC with the AUC_0-6_ is visualized as correlation and Bland-Altman plots (**Figure 3**). With the exception of AUC_0-1_, the R^2^ for the correlation of each normalized reduced interval AUC with the AUC_0-6_ was >0.85. One-sample *t*-tests demonstrated that in all cases the difference between the normalized reduced interval AUC and AUC_0-6_ were not significantly different from 0 (p ≥ 0.76). Linear regression analysis demonstrated that there was no effect of the mean midazolam AUC on the difference in midazolam AUC, indicating a lack of proportional bias within each dataset (p ≥ 0.621). Based on fulfilment of the criteria for acceptance of precision and accuracy, the shortest acceptable sampling interval for assessment of absolute midazolam AUC was 0 to 3 hours. For absolute AUC, precision was also assessed at each data point in the validation dataset (n = 20; 60 data points) as the difference between the normalized reduced interval AUC and the corresponding AUC_0-6_. In all cases the difference between the normalized reduced interval AUC estimates in the AUC_0-3_ and AUC_0-4_ datasets were <20% of the corresponding mean parameter estimate. Similarly, analysis of the correlation of the raw midazolam AUC measured over 0 to 3, 0 to 4, and 0 to 6 h versus the full midazolam AUC (AUC_0-inf_) demonstrated that in all cases the R^2^ for the correlation was greater than 0.875 (**Figure 4**).

**Table 4:** Equations relating reduced sampling duration AUCs to AUC_0-6_ in the training, verification and full (pooled) datasets.

Dataset	Equation			
	AUC_0-1_	AUC_0-2_	AUC_0-3_	AUC_0-4_
Training (n = 30)	y = 3.95x	y = 1.98x	y = 1.47x	y = 1.25x
Verification (n = 60)	Y = 3.96x	Y = 1.97x	Y = 1.45x	Y = 1.22x
Full (n = 90)	y = 3.95x	y = 1.97x	y = 1.46x	y = 1.23x

### Capacity of Reduced Sampling Interval to Define Change in Midazolam AUC Due to Induction

**Figure 5:**
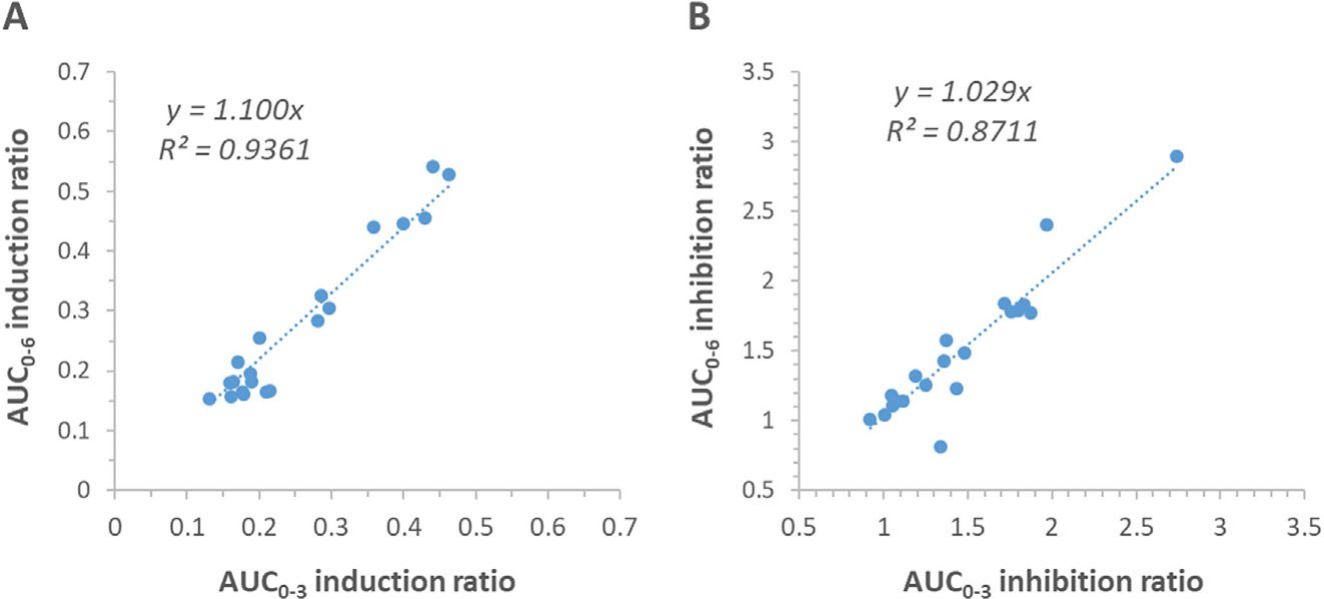
Correlation plots demonstrating the concordance of the change in midazolam AUC due to induction **(A)** and mechanism-based inhibition **(B)** between the normalized 0 to 3 h reduced sampling interval AUC (AUC_0-3_) and AUC_0-6_ in the verification cohort.

Correlation of the AUC_0-3_ (R^2^ = 0.94) and AUC_0-4_ (R^2^ = 0.98) ratios with the AUC_0-6_ ratio fulfilled the criteria of an R^2^ > 0.85, whereas the R^2^ for the correlation of the AUC_0-1_ and AUC_0-2_ ratios with the AUC_0-6_ ratio failed to meet this criteria. One-sample *t*-tests demonstrated that in all cases the difference between the reduced interval AUC ratios and the AUC_0-6_ ratio were not significantly different from 0 (p ≥ 0.062). Linear regression analysis demonstrated that there was no effect of the mean AUC ratio on the difference in the AUC ratio, indicating a lack of proportional bias within each dataset (p ≥ 0.065). Based on fulfilment of the criteria for acceptance of precision and accuracy, the shortest acceptable sampling interval for assessment of induction of midazolam AUC was 0 to 3 h. The correlation of the Day 8/Day 1 (induction) AUC ratio for the AUC_0-3_ reduced sampling interval compared to the AUC_0-6_ ratio is presented in **Figure 5A**. Bland-Altman plots for each reduced sampling interval AUC are presented in **Supplemental Figure 1**.

### Capacity of Reduced Sampling Interval to Define Change in Midazolam AUC Due to Inhibition

Correlation of the AUC_0-3_ (R^2^ = 0.87) and AUC_0-4_ (R^2^ = 0.97) ratios with the AUC_0-6_ ratio fulfilled the criteria of an R^2^ > 0.85, whereas the R^2^ for the correlation of the AUC_0-1_ and AUC_0-2_ ratios with the AUC_0-6_ ratio failed to meet this criteria. One-sample *t*-tests demonstrated that in all cases the difference between the reduced interval AUC ratios and AUC_0-6_ ratio were not significantly different from 0 (p ≥ 0.148). Linear regression analysis demonstrated that there was no effect of the mean midazolam AUC ratio on the difference in midazolam AUC ratio, indicating a lack of proportional bias within each dataset (p ≥ 0.277). Based on fulfilment of the criteria for acceptance of precision and accuracy, the shortest acceptable sampling interval for assessment of induction of midazolam AUC was 0 to 3 h. The correlation of the Day 15/Day 1 (mechanism-based inhibition) AUC ratio for the AUC_0-3_ reduced sampling interval compared to the AUC_0-6_ ratio is presented in **Figure 5B**. Bland-Altman plots for each reduced sampling interval AUC are presented in **Supplemental Figure 2**.

### Comparison of Midazolam AUC_0-3_ With Reported Alternate Metrics

Results described above evaluate the absolute performance of the midazolam AUC_0-3_ with respect to defining midazolam exposure. The capacity of midazolam AUC_0-3_ to define midazolam exposure was also evaluated with respect to comparative performance by reproducing previously proposed metrics in the current validation dataset.

Consistent with the prior analysis of Chaobal and Kharasch (2005), the correlation of single time point midazolam concentrations at 3, 4, and 6 h with midazolam AUC_0-6_ varied between time point and study phases; R^2^ values ranged from 0.357 (6 h control phase) and 0.917 (3 h inhibition phase) (**Supplemental Figures 3A–C**). Correlations of single time point measures with AUC_0-6_ were consistently strongest at the 3 h time point (R^2^ 0.809, 0.879, and 0.917 for baseline, induced, and inhibited phases, respectively), and for the inhibition study phase (R^2^ 0.917, 0.888, and 0.742 for 3, 4, and 6 h time points, respectively). In comparison, the R^2^ vales for the correlation of midazolam AUC_0-3_ with AUC_0-6_ for the control, induction, and inhibition study phases were 0.931, 0.906, and 0.971, respectively (**Supplemental Figure 3D**).

Similarly, consistent with the conclusion of Lee et al. (2006), correlation of single time point OH-midazolam to midazolam ratios at 3, 4, and 6 h with AUC_0-6_ consistently yielded R^2^ values <0.8 for both individual study phases and pooled study phases (not shown).

## Discussion

Here we describe, for the first time, the validation of a 3-h midazolam sampling interval to support the assessment of between-subject variability in CYP3A phenotype across specific sub-populations as well as the evaluation of changes in CYP3A function due to induction and mechanism-based inhibition of this enzyme. The key distinctions between the current analysis and prior analyses (Lee et al., 2006; Katzenmaier et al., 2010; Mueller and Drewelow, 2013; Tai et al., 2013; Masters et al., 2015; Yang et al., 2018) are a) the current study design has allowed for not only the validation of performance under normal, induced and inhibited conditions, but critically also the validation of performance with respect to describing the change in CYP3A function caused by induction and inhibition, and b) the primary focus of the current analysis was to reduce the duration over which samples are collected (i.e. retained rich sampling in the first 2 h), whereas prior analyses have focused on minimizing the number of sample collections (i.e., fewer samples, but at times over a longer period up to 8 h). The robustness of the approach is supported by post-hoc analysis demonstrating that the scaling factor used to normalize the reduced interval AUCs in the training dataset were independently reproducible in the verification and full datasets. Based on these data, a reproducible scaling factor of 1.46 (full dataset value) is proposed to facilitate future normalizations of AUC_0-3_ data to prior or alternate studies where AUC_0-6_ has been measured.

In this regard, prior pooled analyses of three (Kim et al., 2002) and seven (Masters et al., 2015) trials did evaluate the performance of reduced sampling interval AUCs under normal, induced and inhibited conditions, however, data for each set of conditions came from different study cohorts. Critically the use of different cohorts for each set of conditions precluded these prior studies from evaluating performance with respect to the change in CYP3A function caused by induction or inhibition. Similarly, the prior study of Katzenmaier et al. (2010) only considered the performance of reduced sampling interval AUCs under normal conditions. Indeed, the sampling interval proposed by Katzenmaier et al. (2010) explicitly prevents interpretation of the changes midazolam bioavailability (which are predominantly observed as changes in the maximal plasma concentration; C_max_), which, given the low midazolam basal f_g_ of 0.26, is a major factor driving the reduction in midazolam AUC associated with induction of CYP3A (Kapetas et al., 2019). Furthermore, it is worth noting that while the current study validated a longer reduced PK sampling interval compared to Katzenmaier et al. (2010) (3 h versus 2 h), the actual time commitment for study participants and staff following midazolam dosing proposed in the current study is 1 h shorter (3 h versus 4 h) as the sampling interval of Katzenmaier et al. has a 2-h lag phase between midazolam dosing and sample collection. Similarly, consistent with the findings of Lee et al. (2006) and Chaobal and Kharasch (2005), AUC_0-3_ outperformed both single time point midazolam and single time point OH-midazolam to midazolam ratios with respect to defining variability in midazolam AUC_0-6_.

Caution is recommended when extrapolating AUC_0-inf_ based on a measured AUC defining <80% due to a risk of bias and variance associated with the estimation of the elimination rate constant (Purves, 1992). In the cohort analyzed here (n = 30; 90 data points), extrapolation of the midazolam AUC measured over six hours (AUC_0-6_) to infinity accounted for 82%, 83%, and 80% of the AUC_0-inf_ for the baseline, induced, and inhibited study phases, respectively. By comparison, extrapolation of the AUC_0-4_ accounted for only 66%, 68%, and 64% of the AUC_0-inf_ for the baseline, induced, and inhibited phases, respectively. This limitation is important to consider when determination of midazolam apparent oral clearance (CL/F) is the primary variable being considered, as calculation of CL/F requires robust determination of AUC_0-inf_. Under such circumstances the use of a reduced sampling interval AUC may not be appropriate as this strategy does not meet the criteria for extrapolation of AUC_0-inf_. It is worth noting however, that data presented in **Figure 4** support the capacity of reduced sampling intervals measured over 0 to 3 and 0 to 4 h to define >87% of the variability in AUC_0-inf_. Two further limitations that are specific to the current analysis were 1) that while the cohort contained individuals of an appropriate age range for healthy volunteer studies (i.e., 21 to 35 years), the cohort was exclusively male, and 2) the full AUC (AUC_0-inf_) was estimated by extrapolation of the AUC_0-6_ data. However, 1) there is no reason to suspect a gender difference in the performance of a reduced sampling interval AUC given that there is not a statistically significant gender difference observed for the change in midazolam AUC_0-6_ due to induction of CYP3A4 (Kapetas et al., 2019). Similarly, 2) the AUC_0-6_ data accounted for >80% of the full AUC, and thus is likely to provide a robust estimation of this parameter, although full validation of this approach metric should be undertaken in either a prospective study, or by reanalysis of an existing dataset where AUC_0-24_ has been captured.

The reduction in logistical burden associated with reducing the sampling interval for midazolam-based CYP3A phenotyping is likely to have the greatest impact for research performed in academia and clinical sites. Typically, in the pharmaceutical industry, drug-drug interaction (DDI) studies with midazolam are typically designed to assess drug concentration out to 24–48 h to calculate AUC over this PK sampling interval as well as AUC_inf_. Depending on the mechanism of the DDI (inhibition vs induction) and magnitude (none-weak-moderate-strong), such protocols make use of all available data points to calculate midazolam AUC (within assay limits). There may however be industry applications to a reduced sampling interval AUC for midazolam-based CYP3A phenotyping, in terms of reducing blood draws when phenotyping pediatrics, elderly or other vulnerable populations.

A number of recent studies have attempted to remove the requirement to dose a drug probe to assess CYP3A phenotype by measuring either an endogenous small molecule substrate for this enzyme (Wang et al., 1997; Diczfalusy et al., 2011; Bjorkhem-Bergman et al., 2013; Mårde Arrhén et al., 2013), or endogenous protein/nucleic acid biomarker for this enzyme (Rodrigues and Rowland, 2018). While endogenous exosome derived biomarkers (Rowland et al., 2019) appear to represent a promising avenue moving forward, the robust validation of such strategies in both healthy volunteer and patient cohorts require substantial work, thus clinical phenotyping for CYP3A, based on midazolam PK assessment, will likely remain an important tool in both academic and industry research settings for many years.

In conclusion, the current study demonstrates a robust concordance between midazolam AUC calculated by either 0 to 3 and 0 to 6 h PK sampling intervals in terms of both the measurement of between subject variability and changes in midazolam exposure due to induction and mechanism-based inhibition of CYP3A4. On this basis, it is proposed that assessment of a 0 to 3hr midazolam AUC (AUC_0-3_) represents a viable strategy to reduce the logistical burden associated with the assessment of CYP3A phenotype and perturbations of phenotype caused by CYP3A induction or mechanism-based inhibition.

## Data Availability Statement

All datasets generated for this study are included in the manuscript/**Supplementary Files**.

## Ethics Statement

The study protocol was approved by the Southern Adelaide Clinical Human Research Ethics Committee (SAHREC 11.15), and written informed consent was obtained from each participant prior to study enrolment. The study was conducted according to the principles stated in the Declaration of Helsinki, was compliant with CPMP/ICH/135/95 GCP standards, and is registered with the Australian New Zealand Clinical Trials Registry (ACTRN 12614001289606).

## Author Contributions

Designed the research: MD, ADR, MV, MS, and AR. Sample collection and analysis: MD and AR. Data analysis: AK, AH, and AR. Wrote the manuscript: MD, AK, AH, ADR, MV, MS, and AR.

## Funding

This study was supported by a project grant [1100179] from the National Health and Medical Research Council of Australia. AR is supported by a Beat Cancer Mid-Career Fellowship from Cancer Council, Australia. AH is supported by a Postdoctoral Fellowship from the National Breast Cancer Foundation, Australia.

## Conflict of Interest

MS and AR are recipients of industry funding for investigator initiated clinical trials from Pfizer Inc. Pfizer Inc provided support with study design and preparation of the manuscript. ADR and MV are employees and stock holders of Pfizer Inc. 

The remaining authors declare that the research was conducted in the absence of any commercial or financial relationships that could be construed as a potential conflict of interest.
