# Supplementary figures and images for: Validation of a 3-h Sampling Interval to Assess Variability in Cytochrome P450 3A Phenotype and the Impact of Induction and Mechanism-Based Inhibition Using Midazolam as a Probe Substrate
Source: Front Pharmacol. 2019 Sep 27;10:1120. doi: 10.3389/fphar.2019.01120 (PMC6777419; doi:10.3389/fphar.2019.01120)

**Supplemental Figure 1**


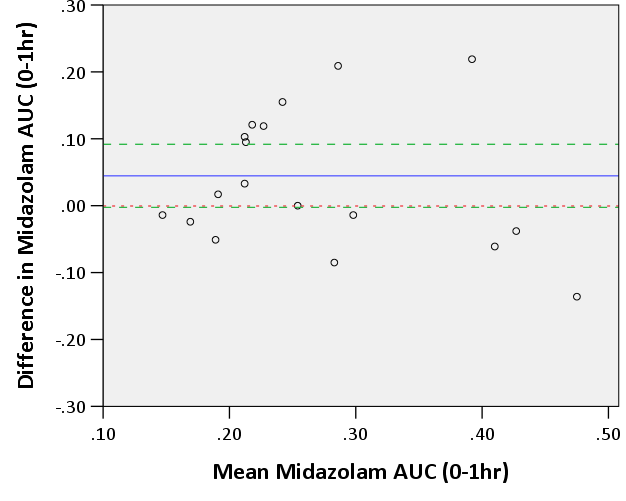

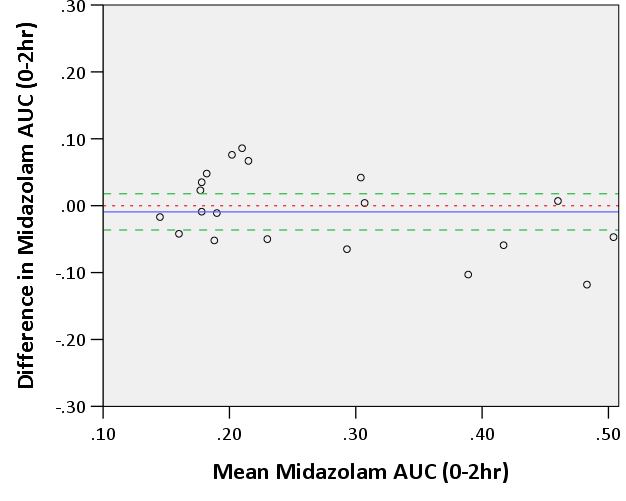


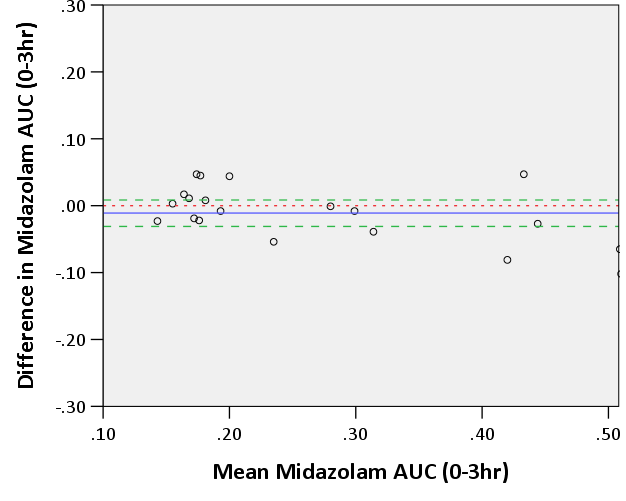

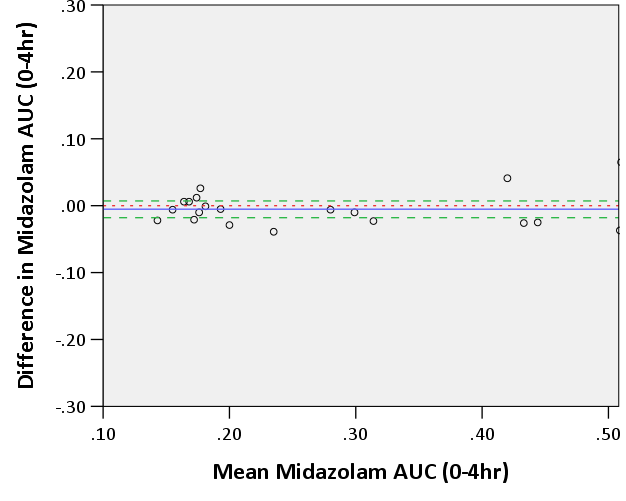


**Supplemental Figure 2**


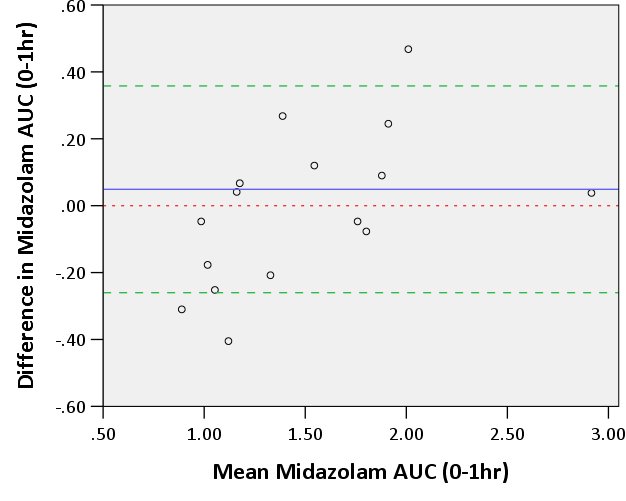

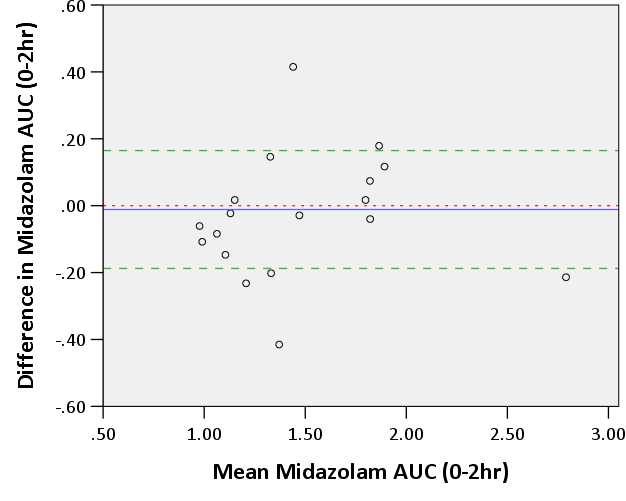

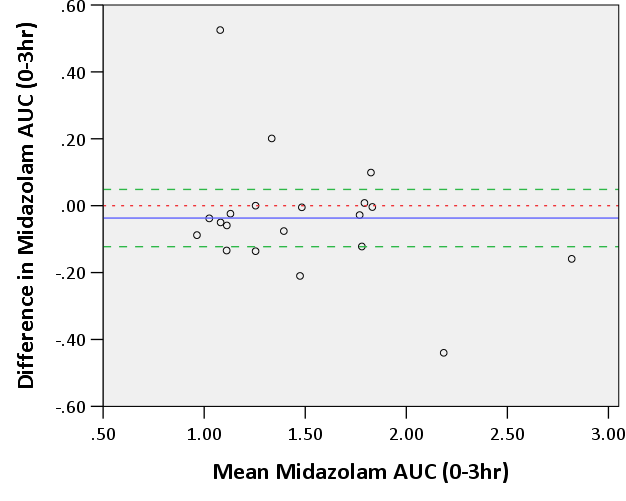

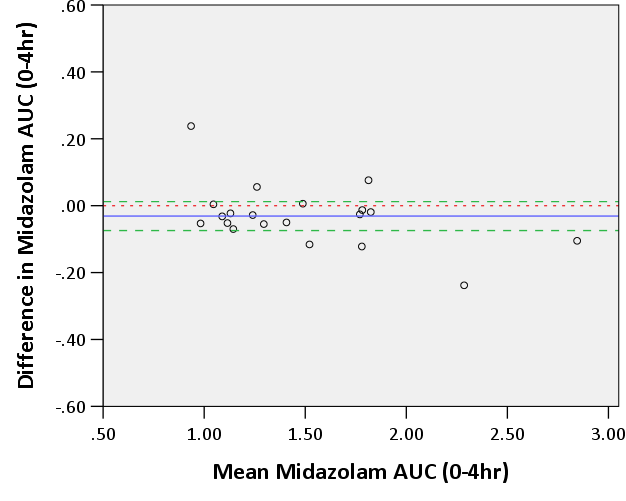


**Supplemental Figure 3**


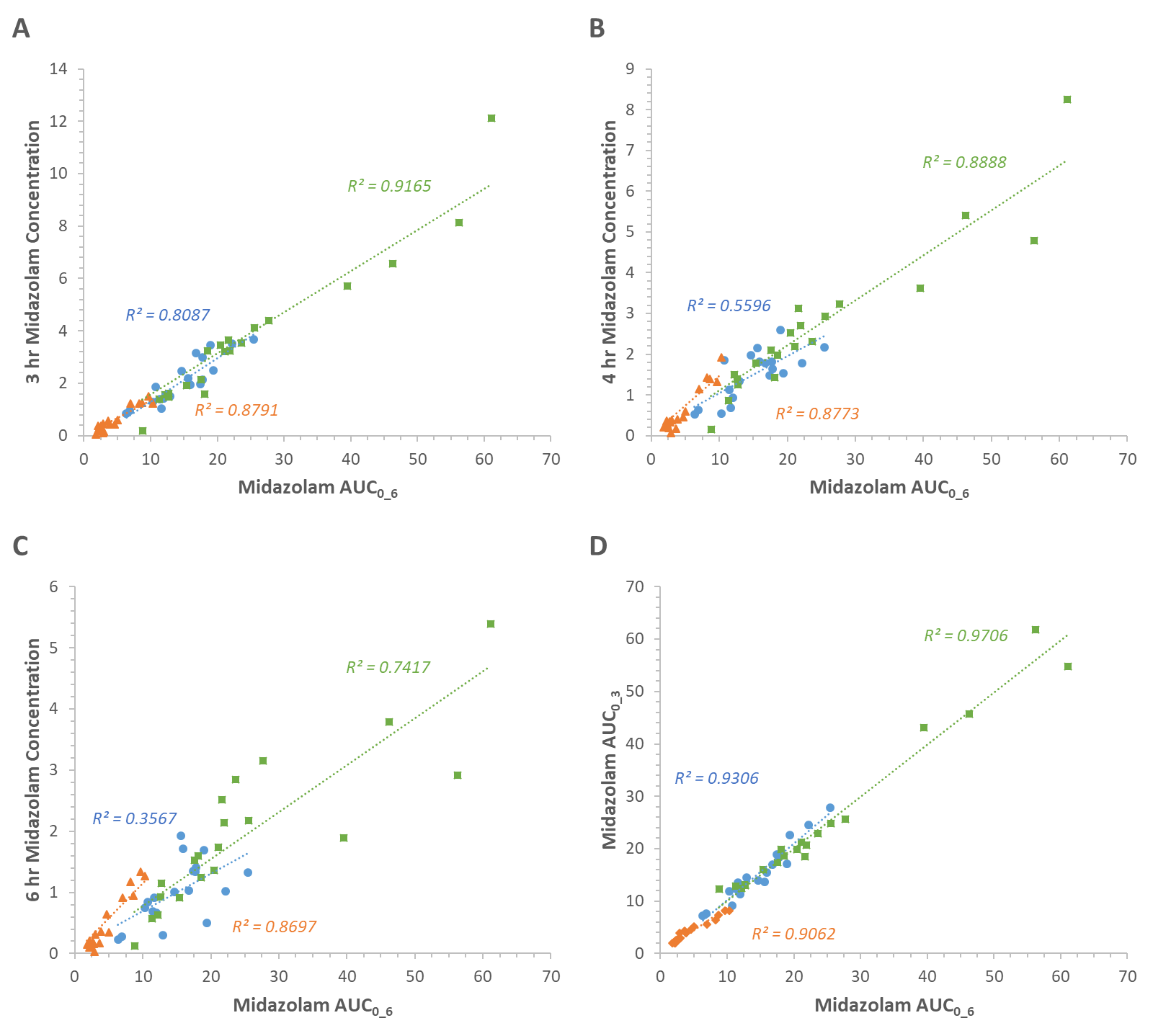

Supplement: Supplementary file 1 [file DataSheet_1.docx]
